# Photoacoustic/Ultrasound/Optical Coherence Tomography Evaluation of Melanoma Lesion and Healthy Skin in a Swine Model

**DOI:** 10.3390/s19122815

**Published:** 2019-06-24

**Authors:** Karl Kratkiewicz, Rayyan Manwar, Ali Rajabi-Estarabadi, Joseph Fakhoury, Jurgita Meiliute, Steven Daveluy, Darius Mehregan, Kamran (Mohammad) Avanaki

**Affiliations:** 1 Department of Biomedical Engineering, Wayne State University, Detroit, MI 48201, USA; karl.kratkiewicz@wayne.edu (K.K.); r.manwar@wayne.edu (R.M.); 2 Dr. Phillip Frost Department of Dermatology and Cutaneous Surgery, University of Miami Miller School of Medicine, Miami, FL 33136, USA; arajabi@med.miami.edu; 3 Wayne State University School of Medicine, Detroit, MI 48201, USA; jfakhour@med.wayne.edu (J.F.); dmehregan@wayne.edu (D.M.); 4 EKSPLA USA, Bozeman, MT 59715, USA; jurgita@altosphotonics.com; 5 Department of Neurology, Wayne State University School of Medicine, Detroit, MI 48201, USA; sdaveluy@med.wayne.edu; 6 Barbara Ann Karmanos Cancer Institute, Detroit, MI 48201, USA

**Keywords:** photoacoustic imaging, cancer imaging, skin imaging, ultrasound, optical coherence tomography, melanoma, swine melanoma model

## Abstract

The marked increase in the incidence of melanoma coupled with the rapid drop in the survival rate after metastasis has promoted the investigation into improved diagnostic methods for melanoma. High-frequency ultrasound (US), optical coherence tomography (OCT), and photoacoustic imaging (PAI) are three potential modalities that can assist a dermatologist by providing extra information beyond dermoscopic features. In this study, we imaged a swine model with spontaneous melanoma using these modalities and compared the images with images of nearby healthy skin. Histology images were used for validation.

## 1. Introduction

Melanoma is the most dangerous form of skin cancer. Every year about 100,000 new cases of melanoma are diagnosed in the United States [1]. This incidence is doubled every 10 years [2]. In recent years, due to climate change, the incidence of melanoma has increased markedly, especially in fair-skinned populations [3]. The lifetime risk of developing melanoma for persons born in the year 2014 is 1 in 50 [4]. The survival rate from melanoma is about 98% before metastasis, but drops to about 20% with distant metastasis [4]. Thus, early detection of melanoma is critical to preventing mortality.

Lesions suspicious for melanoma should undergo excisional biopsy; however, performing a biopsy creates pain, scarring, and disfigurements [5,6,7]. Biopsy also creates anxiety for the patients while they are waiting for the histopathology results. Partial (incisional) biopsies can lead to sampling error and inaccurate assessment of the lesion [8]. Thus, a non-invasive imaging technique that could discern melanoma from healthy tissue while visualizing tumor depth in real-time would allow for accurate diagnosis and surgical planning. 

Numerous non-invasive imaging modalities are under investigation to aid in the early detection of melanoma (Table 1). High-frequency ultrasound (US) can measure melanoma depth, but the image contrast is poor due to the small difference in the acoustic impedance between melanoma and the surrounding tissues [9,10,11,12]. Optical methods, such as confocal microscopy (CM) [13] and optical coherence tomography (OCT) [14,15,16,17,18,19,20,21,22,23], employ the scattering coefficient as a contrast to facilitate melanoma detection, but the penetration depth is limited. Other methods include multispectral imaging (MSI) [24,25,26], hyperspectral imaging (HSI) [27,28,29], Raman spectroscopy (RS) [30,31,32], and electrical impedance imaging (EI) [33,34,35]. There are other methods to study skin that have not been fully explored, such as the one in [36]. 

**Figure 1:**
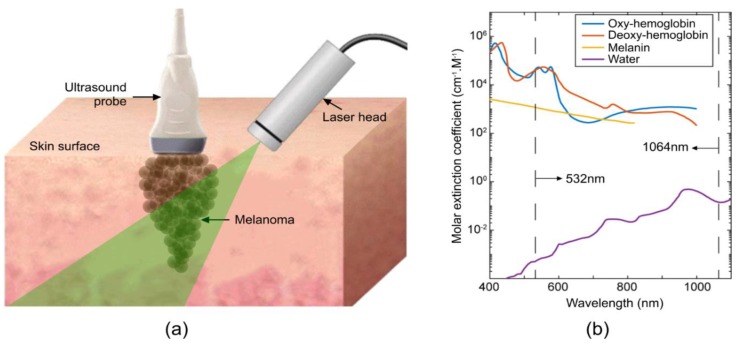
Principle of photoacoustic imaging. (**a**) Schematic of photoacoustic imaging setup for the acquisition of images from swine skin. (**b**) Optical absorption spectrum for most abundant photoacoustic absorbers in the skin with dashed lines showing wavelengths used in this study. Left: 532 nm; right: 1064 nm.

Photoacoustic (PA) tomography is a non-invasive, non-ionizing, and functional imaging technique that can be used at the microscopic (photoacoustic microscopy (PAM)) [43,44,45] or macroscopic (photoacoustic computed tomography (PACT)) scales [46,47,48,49,50,51]. This kind of imaging combines the contrast of optical imaging with the spatial resolution of ultrasound. A nanosecond pulsed laser deposits energy onto a light-absorbing sample (such as the skin), causing a local temperature to increase, with subsequent thermal expansion through the thermoacoustic effect [52,53,54,55,56]. The thermal expansion yields a localized pressure increase, resulting in the formation of ultrasound waves that are detected by an ultrasound transducer to produce an image [57,58,59,60]. Various ultrasound transducer configurations can be designed for optimal imaging of the specified target [46,61,62,63,64,65,66,67]. This can be utilized to distinguish specific markers between healthy and melanoma tissue, as seen in Figure 1. The limitations of the other techniques provided in Table 1 can be solved through the use of a combined US/PA system to discern structural information, provided by US, with molecular information, provided by PA. For example, the sensitivity to hemoglobin may be used to image angiogenesis in the growing tumor [68]. PA has previously been studied in cutaneous melanoma [69,70,71,72,73,74], demonstrating its ability to delineate melanoma tumor depth in animal models [69] and humans [70]. Currently, the differences between melanoma and healthy skin on large animals with spontaneous melanoma using photoacoustic imaging have not been elucidated.

## 2. Materials and Methods

**Figure 2:**
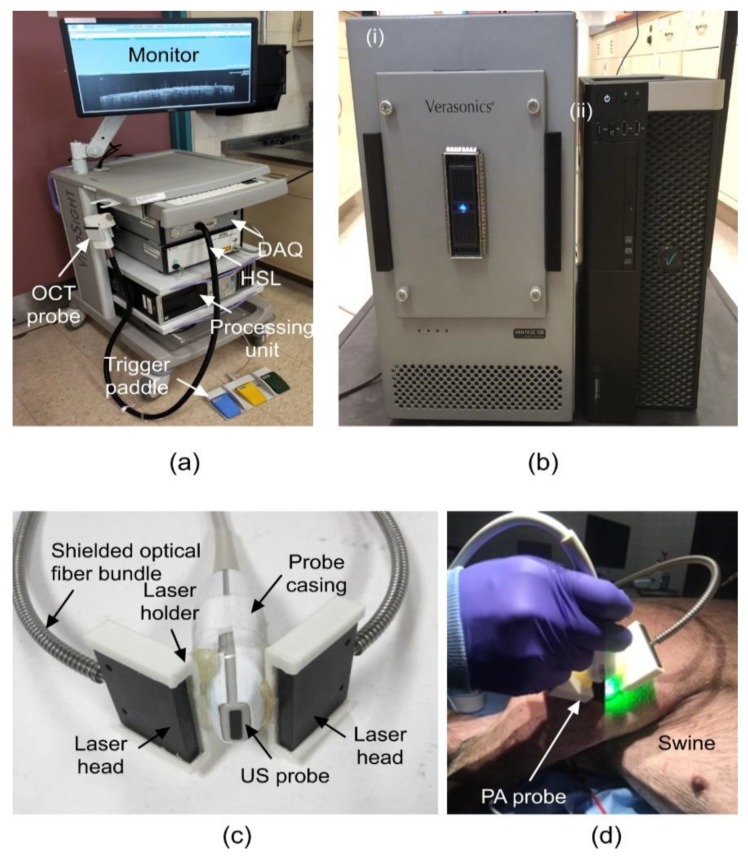
Ultrasound (US)/photoacoustic (PA) system components. (**a**) Optical coherence tomography (OCT) system. (**b**) US/PA DAQ, processing, and storage units, (i) Vantage 128 DAQ system, and (ii) processing unit. (**c**) US/PA probe specifications. (**d**) US/PA probe in use on swine melanoma lesion. DAQ: Data acquisition unit, HSL: High-speed swept-source laser.

We developed a US/PA imaging system comprising a Vantage 128 system (Verasonics, inc., Kirkland, WA, USA) with attached L22-14v ultrasound transducer (Philips, Amsterdam, Netherlands). The transducer has a central frequency of 18.5 MHz with 65% bandwidth, making a resolution of the order of ~200 µm. The data acquisition was triggered by a Q-switched Nd:YAG laser (NL231-50-SH, EKSPLA, Vilnius, Lithuania) that was used to illuminate the skin tissue. Upon opening of the laser Q-switch, a trigger was sent to the Verasonics system to initiate the receive mode of the transducer for photoacoustic pressure wave detection. A novel bifurcated fiber bundle was manufactured by Fiberoptics Technology inc., Pomfret, CT, USA, and attached in a 3D-printed housing for homogeneous illumination of the imaging plane as seen in Figure 2. The laser energy was controlled with an attenuator to confirm the maximum energy deposited to be below the American National Standards Institute (ANSI) limits of 20 mJ/cm^2^ for 532 nm and 100 mJ/cm^2^ for 1064 nm [75]. The user interface was coded in the MATLAB software. In between photoacoustic frames, the script prompted a frame of ultrasound as well as for simultaneous US and PA image capture [57,59,60]. The components of the US/PA system can be seen in Figure 2.

The OCT system used in this study (Figure 2a) was a multi-beam, Fourier-domain, swept-source OCT (Vivosight, Michelson Diagnostic TM Inc., Kent, UK) with a central wavelength of 1305 ± 15 nm. The lateral and axial resolution of our system was 7.5 µm and 10 µm, respectively. The 10-kHz sweep rate determined the time to generate one reflectivity profile. The penetration depth of the system was measured as 1.5 mm in healthy human skin [76]. This OCT system was based on multi-beam technology, similar to the technology used in dynamic focus OCT [77], in which four 0.25 mm wide consecutive confocal gates were combined to provide a total confocal gate of 1 mm. Utilizing the multi-beam technology, the images obtained from the four channels were averaged. In OCT, the reflectivity profile was termed as an axial scan (A-scan or A-line). By grouping together several A-lines for different transversal positions of the incident beam on the sample, a cross-section image or a B-scan was generated [78]. The images obtained with this OCT system were B-Scan images with a size of 6 mm × 2 mm and software inferred C-scan images with a size of 6 mm × 6 mm. 

**Figure 3:**
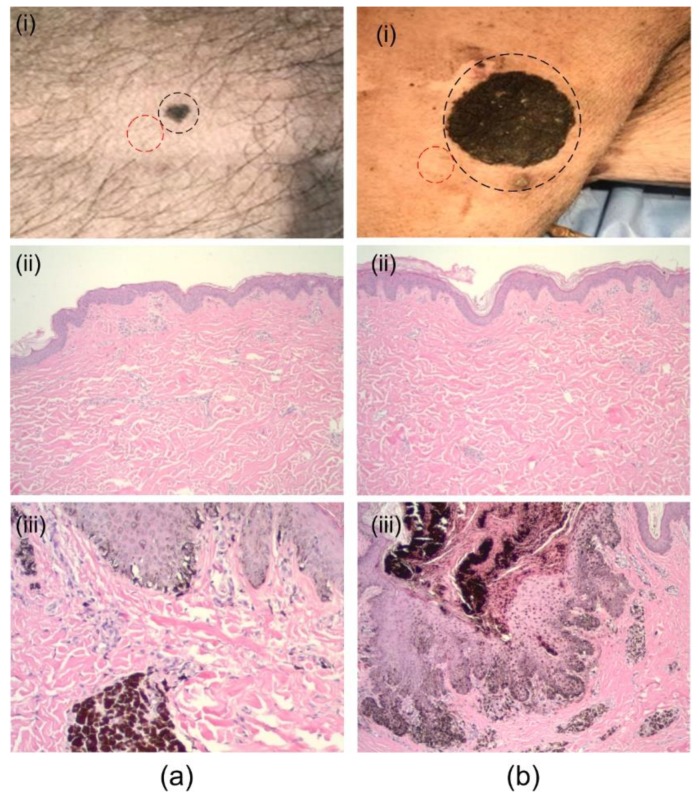
Imaged suspect lesions. (**a**) (i) Abdominal, dark-brown pigmented plaque with irregular border confirmed as melanoma (black-circle), (ii) histology of nearby healthy skin (red-circle), and (iii) histology of the suspect lesion. (**b**) (i) Flank, large dark-brown plaque confirmed as melanoma (circled), (ii) histology of nearby healthy skin, and (iii) histology of the suspect lesion.

We conducted studies in a melanoma swine model (Sinclair Bio-Resources, LLC, Columbia, MO, USA) with histologically verified melanoma and adjacent normal skin (Figure 3). The swine melanoma model was used because of the similarities between human and porcine skin [79,80], particularly the epidermal structure and thickness [81,82,83,84]; the epidermal/dermal junction is also similar to humans. 

For each imaging session, the swine model was sedated with 4.4 mg/kg of telazol and 2.2 mg/kg of xylazine, i.m. administered, prior to the experiment. Ketamine/diazepam was administered via the catheter to effect in order to induce general anesthesia and permit endotracheal intubation, and isoflurane was administered using a precision vaporizer and ventilator. A rumen tube was placed orally to permit removal of gastric contents. The eyes were lubricated with a petrolatum-based product. Heart and respiratory rates, indirect blood pressure, pulse oxygenation, and end tidal CO_2_ were monitored throughout the procedure. Hair on the regions of interest was removed with a shaving clipper and the site was scrubbed three times, alternating between Betadine scrub and alcohol. There were two regions of interest (ROIs): A small lesion on the abdomen and a large lesion on the flank (Figure 3) were imaged with both the US/PA and the OCT system. Healthy skin adjacent to the suspect lesions was also imaged for comparative assessment. US images of all lesions were acquired followed by PA images taken at both 532 nm and 1064 nm. Biopsies were then taken from each lesion.

## 3. Results and Discussion

**Figure 4:**
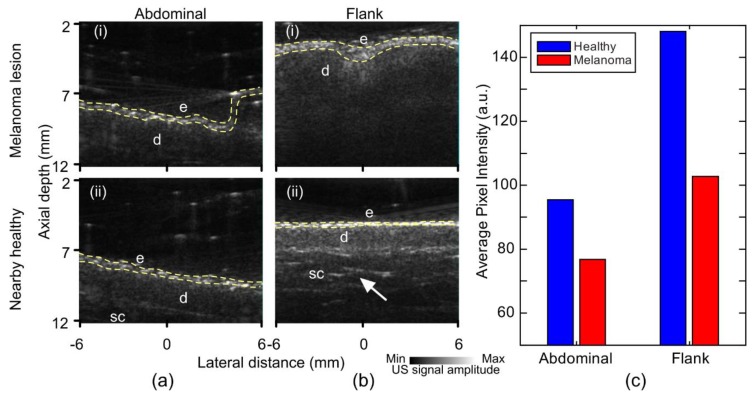
Ultrasound images of melanoma lesion and nearby healthy skin. (**a**) Abdominal: (i) Lesion, (ii) nearby healthy. (**b**) Flank: (i) Lesion, (ii) healthy. (**c**) Bar chart of average pixel intensity from epidermal region of US images. E: Epidermis, d: Dermis, sc: Subcutaneous tissue. Fibrotic septa (arrows), epidermal layer pixels (yellow dashes).

Ultrasound images of both melanoma and nearby healthy skin with the annotations of different regions are shown in Figure 4. It was evident that the melanomas had a dimmer epidermis signal compared to the nearby healthy tissue (Figure 4), implying a reduced US echo from this layer that could be caused by a reduced impedance mismatch between the epidermis and dermis. The lowered melanoma epidermal signal was unexpected, as the density of melanoma cells was greater than healthy tissue, which should result in a greater echo from the lesion. We expect that the melanoma epidermis was dimmer due to irregularity of rete ridges, resulting in major scattering of the acoustic waves. The echogenicity of the two tissues was different. The signal was quantified through the average pixel value of the image and demonstrated in Figure 4c with averaged pixels within the yellow dashes of Figure 4a,b. Further, the dermis was more visible in the healthy tissue, allowing the visualization of fibrotic septa in the dermis of the healthy tissue images. The decreased melanoma dermis signal was due to the disruption of normal tissue architecture from the invasion of melanocytes, resulting in the loss of a clear boundary between the dermis and subcutaneous layers.

**Figure 5:**
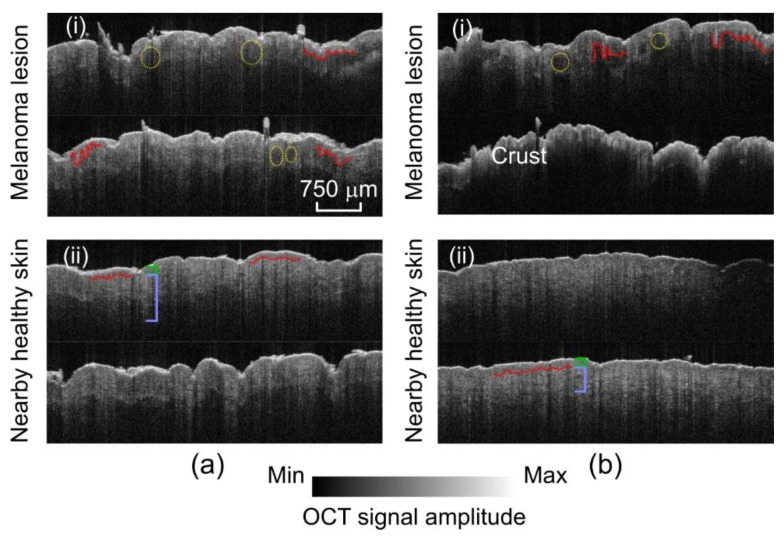
OCT images of melanoma lesion and nearby healthy skin. (**a**) Abdominal: (i) Lesion and (ii) healthy. (**b**) Flank: (i) Lesion and (ii) healthy. Melanomas demonstrate disorganization and thickening of the epidermis, larger rete ridges, an obscured dermal–epidermal junction (DEJ), and dermal tumor nests. Yellow circles: Dermal nests of melanocytes. Red lines: Dermal–epidermal junction. Green brackets: Epidermis. Light blue brackets: Dermis.

OCT imaging was conducted using a triaxial holder to maintain the OCT probe perpendicular to the sample surface. A total of 170 images in the free-run mode of the OCT were collected. In Figure 5, two slides of each lesion and its nearby healthy region are shown. The melanomas displayed a thickened epidermis with more disordered architecture, as also demonstrated in [17]. Rete ridges in melanoma were larger and broader. There were large melanoma nests in the superficial dermis which, combined with the irregularity of the rete ridges, made visualization of the dermal-epidermal junction (DEJ) [85,86] more difficult. These results are consistent with the findings in the literature [87,88,89,90,91,92,93,94,95]. 

**Figure 6:**
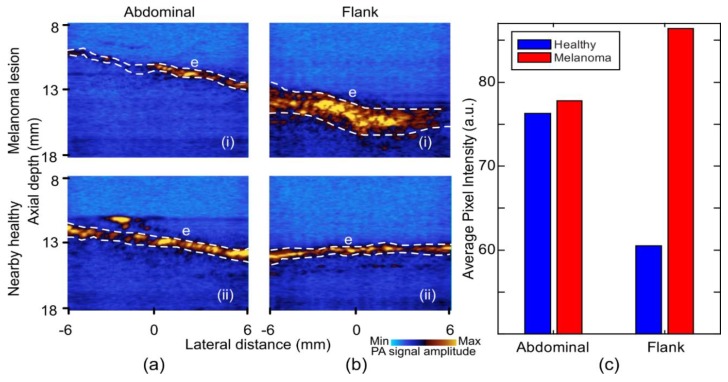
Photoacoustic images of melanoma lesion and nearby healthy skin at 532 nm illumination wavelength. (**a**) Abdominal: (i) Lesion and (ii) healthy. (**b**) Flank: (i) Lesion and (ii) healthy. (**c**) Bar chart of average pixel intensity from epidermal region in the PA images of 532 nm. E: Epidermis. Epidermal pixels (white dashes). The PA signal was increased in the melanoma, highlighting the increase in melanin.

Photoacoustic images acquired at 532 nm wavelength are shown in Figure 6. The main difference between the melanoma and the healthy tissue at 532 nm wavelength was the PA signal strength from the epidermis. Figure 6c shows the average pixel intensity of the epidermal region at 532 nm photoacoustic imaging of both lesions and nearby healthy skin. The flank melanoma in Figure 6b(i), appeared to produce a stronger photoacoustic signal than the healthy tissue at this wavelength, most likely due to the presence of an increased amount of melanin in the epidermis and, thus, higher absorption. In contrast, in the healthy skin a low and uniform photoacoustic signal can be seen in the epidermis, which corresponded to normal melanin distribution in the epidermis (Figure 6a(ii),b(ii)).

**Figure 7:**
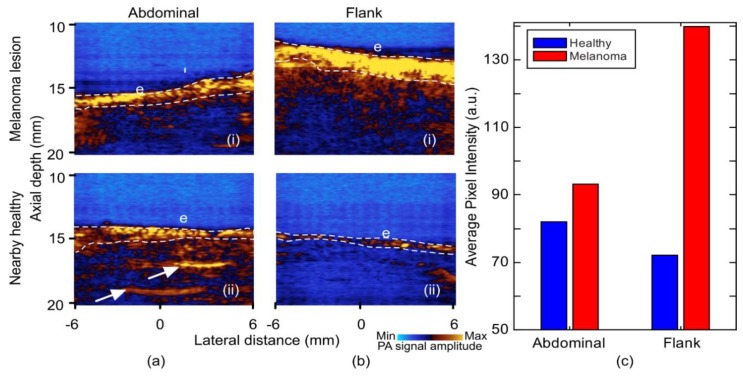
Photoacoustic images of melanoma lesion and nearby healthy skin at 1064 nm illumination wavelength. (**a**) Abdominal: (i) Lesion and (ii) healthy. (**b**) Flank: (i) Lesion and (ii) healthy. (**c**) Bar chart of average pixel intensity from epidermal region at 1064 nm images. E: Epidermis. Fibrotic septa (arrows); averaged epidermal pixels (white dashes).

Photoacoustic images acquired at 1064 nm wavelength with annotations are shown in Figure 7. At this wavelength, it appeared that the photoacoustic signal from the epidermis had a higher intensity from melanoma as compared to the normal skin (Figure 7a(i),b(i)). Image quantification extracted from the average epidermal signal strength is shown in Figure 7c, with averaged pixels within the white dashes of Figure 7a,b. This signal continued into the superficial dermis in melanoma. Furthermore, with the deeper penetration of light from the longer wavelength, fibrotic septa can also be seen in healthy tissue (Figure 7a(ii)) similar to those seen in the ultrasound images. The difference in the signal strength between the healthy regions was due to the change in gain settings to avoid any image saturation from the flank region.

In Table 2, we have summarized the capability, advantages, and limitations of each of the imaging modalities used for this study.

Although the US images showed structural differences between the melanoma and healthy skin, this modality was unable to identify micron-level morphological changes in the skin due to melanoma. Therefore, US can provide information on the size and shape of a lesion, but cannot provide details regarding the diagnosis or malignant potential. Higher frequency US probes could enable cellular imaging while maintaining a sufficient penetration depth and provide more specificity for diagnosis. OCT helped to display disordered architectural organization in rete ridges and the dermal-epidermal junction, and helped visualize the infiltrative nature of the tumor [96]. With photoacoustic imaging (PA), we were able to extract information related to melanin content in the tumors through epidermal signal strength. As shown in Figure 1b, 532 nm illumination will have the greatest PA signal from high absorption of oxy- and deoxy-hemoglobin with some signal from lower absorption of melanin and minimal absorption of water. This wavelength may allow for the staging of angiogenesis in the melanoma lesion. In Figure 1b, 1064 nm illumination had comparable absorption by oxy-, deoxy-hemoglobin, and melanin, and minimal absorption from water. This wavelength may show changes in the melanin content in the lesion. Exact contributions to photoacoustic signals from multiple optical absorbers require the use of photoacoustic spectroscopy, i.e., semi-simultaneous imaging using several wavelengths [96]. As we did not have access to a broad-spectrum tunable laser source for this study, only total absorption was explored in our images. Moreover, the oxygen uptake rate of cancerous tissue was higher as compared to normal skin, which can be measured in the PA images. PA was also capable of detecting the bottom boundary of the tumor. Limitations observed in each of these imaging modalities led us to believe that a combined US/PA/OCT imaging technique could help clinicians in the diagnosis of melanoma.

## 4. Conclusions

There is a need for a non-invasive in vivo imaging for rapid diagnosis of melanoma. We presented the results of three imaging modalities—ultrasound, optical coherence tomography, and photoacoustic imaging—to study the image features of melanoma compared to those in nearby healthy skin tissue and evaluated their clinical capability. It can be seen that there are subtle differences between lesioned and healthy tissues in the US: A stronger signal from the epidermis and dermis, and a presence of fibrotic septa in healthy tissue, whereas comparatively weaker US intensity and no presence of fibrotic septa was observed at the melanoma site. We were able to visualize the micron-level morphological differences between the melanoma and healthy tissues for thin lesions using OCT. Melanoma lesions displayed a more disordered architectural organization in OCT images, rete ridges with a broadened shape, and a less defined dermal–epidermal junction due to the infiltrative nature of the tumor growth and the irregularity of rete ridges. In PA imaging, the use of 532 nm wavelength illumination demonstrated a difference in the epidermal signal, with melanoma lesions being stronger most likely due to greater pigmentation. The 1064 nm illumination presented a stronger epidermal signal; however, it also provided the information regarding the presence of fibrotic septa in the healthy tissue due to deeper illumination penetration. Our findings are not necessarily specific to melanoma since we did not have access to benign nevi lesion in the same animal for a fair comparison. Further, the melanomas imaged were thick with large nests of melanocytes. These observations may not pertain to imaging early, thin melanoma. More research is necessary to determine which findings are specific to melanoma and whether or not they can be used to distinguish melanoma and benign melanocytic tumors. 

Combining our observations, healthy skin demonstrated fibrotic septa in the subcutaneous region in US, thinner epidermis with patterned structure in OCT, and a lower average PA signal intensity from epidermis. In melanoma, we observed a reduced dermal signal in US, a stronger epidermal photoacoustic signal intensity, and larger and broader rete ridges in OCT.

This pilot study evaluated differences between melanoma and healthy tissue and we are looking to perform evaluations on a larger number of samples to confirm the trends seen in the images provided in this study.

## Figures and Tables

**Table 1:** Limitations of non-invasive melanoma imaging methods.

Imaging Modality	Limitation	Clinical Problem
**Dermoscopy (D)** [37,38,39]	Depends on appearance of classic dermoscopic features. Requires training to provide advantage over clinical examination	Failure to recognize melanomas that lack specific dermoscopic criteria
**Multispectral imaging (MSI)** [24,25,26]	Data is projected onto the same plane	Obscures depth information of melanoma
**Reflectance confocal microscopy (RCM)** [40,41,42]	Limited field of view and penetration depth	Unable to determine depth of invasion
**High-frequency ultrasound (HFS)** [9,10,11,12]	Low specificity	Inability to diagnose type of tumor
**Raman spectroscopy (RS)** [30,31,32]	Analysis of chemical composition of melanoma	Lacks depth discrimination similar to multispectral imaging
**Electrical impedance imaging (EI)** [33,34,35]	Analysis of electrical impedance spectrum of lesion	Cannot distinguish nevi from melanoma
**Optical coherence tomography (OCT)** [14,15,16,17]	Limited penetration depth	Unable to determine depth of invasion

**Table 2:** Advantages and disadvantages of US, OCT, and PA imaging for melanoma imaging.

Imaging Modality	Imaging Capability	Advantage	Limitations	Findings in Lesional Area
**US**	Structural–morphology of different structures in skin	Penetration depth (up to 2 cm)	Insufficient resolution even using high-frequency probes	(i) Weaker signal from epidermis and dermis (ii) Absence of fibrotic septa
**OCT**	High-resolution morphology	Superior resolution (1~10 µm depending on the configuration of OCT)	Limited penetration depth (~1.5 mm)	(i) Broadened shape of rete ridges (ii) Less defined dermal–epidermal junction
**PA**	Vascular pattern and oxygenation maps	Multispectral imaging	Insufficient resolution for cellular imaging	(i) Stronger signal from epidermis layer
